# Airtight storage of moist wheat grain improves bioethanol yields

**DOI:** 10.1186/1754-6834-2-16

**Published:** 2009-08-20

**Authors:** Volkmar Passoth, Anna Eriksson, Mats Sandgren, Jerry Ståhlberg, Kathleen Piens, Johan Schnürer

**Affiliations:** 1 Uppsala Biocenter, Department of Microbiology, Swedish University of Agricultural Sciences, SE-750 07 Uppsala, Sweden; 2 Chematur Engineering, SE-691 27 Karlskoga, Sweden; 3 Uppsala Biocenter, Department of Molecular Biology, Swedish University of Agricultural Sciences, SE-751 24 Uppsala, Sweden; 4 Laboratory for Protein Biochemistry and Biomolecular Engineering, Department of Biochemistry and Microbiology, Ghent University, 9000 Ghent, Belgium

## Abstract

**Background:**

Drying is currently the most frequently used conservation method for cereal grain, which in temperate climates consumes a major part of process energy. Airtight storage of moist feed grain using the biocontrol yeast *Pichia anomala *as biopreservation agent can substantially reduce the process energy for grain storage. In this study we tested the potential of moist stored grain for bioethanol production.

**Results:**

The ethanol yield from moist wheat was enhanced by 14% compared with the control obtained from traditionally (dry) stored grain. This enhancement was observed independently of whether or not *P. anomala *was added to the storage system, indicating that *P. anomala *does not impair ethanol fermentation. Starch and sugar analyses showed that during pre-treatment the starch of moist grain was better degraded by amylase treatment than that of the dry grain. Additional pre-treatment with cellulose and hemicellulose-degrading enzymes did not further increase the total ethanol yield. Sugar analysis after this pre-treatment showed an increased release of sugars not fermentable by *Saccharomyces cerevisiae*.

**Conclusion:**

The ethanol yield from wheat grain is increased by airtight storage of moist grain, which in addition can save substantial amounts of energy used for drying the grain. This provides a new opportunity to increase the sustainability of bioethanol production.

## Background

In temperate climates, harvest of cereal grain must often be done at high moisture content as the vegetation period is rather short. This requires high amounts of energy for drying to enable safe storage of the harvested material and avoid mould growth. In Sweden, hot-air drying is often the process during grain production that consumes the highest proportion of input energy, that is, up to 60% [1]. With regard to biofuel production from cereal grains, there are concerns about the energy balance and sustainability of the currently established processes. A recent study showed that the net output of energy in bioethanol production was rather small when using corn as raw material [2]. Substantial improvements of the energy balance of a bioethanol production process can only be achieved by an optimisation of all the partial processes involved. In regions with a temperate climate, a reduction in energy demand for the storage of the raw material will have a large impact on the energy balance. Due to increasing energy prices, this would also substantially decrease the production costs and thus improve the economic viability of the whole process.

During recent years, we have investigated an alternative storage method for cereals, where moist feed grain is stored in an airtight system. Long-term storage stability, even with temporary air leakages, can be ensured by the addition of the preservative yeast *Pichia anomala*. This yeast has a considerable antifungal activity, and efficiently prevents the growth of moulds on moist grain stored under conditions of restricted air access [3,4]. On farms, airtight storage of feed cereals is achieved by packing the grain either in silos [5], or large plastic tubes with a diameter of 2 m and a length of up to 100 m. Due to the metabolic activity of the grain, and the micro-organisms on the grain, residual oxygen in the system is consumed and carbon dioxide is formed, inhibiting the growth of moulds and other aerobic micro-organisms. The system where the grain is packed into plastic tubes is increasingly used in farms, because it is flexible, technically easy to handle and energy saving [6]. It seems possible that the airtight grain storage system can be adapted for bioethanol production.

However, the use of stored moist grain inoculated with *P. anomala*, for the production of bioethanol has not been tested yet. As starch cannot be fermented to ethanol by the traditional fermentation yeast *Saccharomyces cerevisiae*, the starch in the grains must first be degraded to fermentable sugars, glucose and maltose, by enzymatic degradation before fermentation. In the alcohol industry, the use of enzymes for the production of fermentable sugars from starch is well established and two groups of enzymes, endoamylases and exoamylases, are mainly used [7]. Endoamylases cleave α-1,4 glycosidic bonds present in the inner part of the amylose or amylopectin chain [8], while exoamylases, such as glucoamylase, cleave α-1,4 and α-1,6 glycosidic bonds. They act on the outer glucose residues of amylose or amylopectin and produce only glucose or maltose [9]. The structure of starch may differ between dry and moist wheat [10], which might influence the activity of the starch-degrading enzymes during the pre-treatment. The starch for the ethanol production might be better accessible for the endo/exo-amylase mixture if the grain cell structures are partially degraded before the enzymatic treatment. Thus, an addition of cellulose and pectin-degrading enzymes before the amylase treatment may also enhance the ethanol yield obtained from the grains.

The biocontrol yeast may in itself also have an impact on the enzyme activity or the fermentability of the material. It may consume fermentable sugars or nutrients that are required for the fermentation yeast to efficiently produce ethanol. Moreover, it has been shown that non-*Saccharomyces *yeasts can outcompete *S. cerevisiae *in industrial alcohol fermentations [11,12]. *P. anomala *is rather robust towards environmental stress [3], thus it is not unlikely that some cells would survive the pre-treatment of the grain material and disturb the ethanol production process.

In this study we used airtight stored moist wheat grain for ethanol production to test the impact of this alternative storage technique on the ethanol production yield and the stability of the fermentation process. In addition, we investigated the impact of using enzymes degrading structural polysaccharides on the total ethanol yield.

## Results

### Impact of the storage system on ethanol yield

To test the impact of airtight storage of cereal grain on ethanol production from the grain, storage was simulated on a laboratory scale. Moist wheat (30% water content) was stored in airtight test tubes with a simulated air leakage [4]. A sub-set of these test tubes was inoculated with the biocontrol yeast, *P. anomala *J121. The other tubes were not inoculated to estimate an eventual impact of the biocontrol yeast on the subsequent enzymatic pre-treatment and fermentation. After 4 weeks incubation, 50% of the tubes without yeast inoculation showed substantial mould growth. These tubes were not included in further analyses. No mould growth was observed in tubes inoculated with *P. anomala*. These results showed that it was necessary to include the biocontrol yeast for grain conservation. Farm grain with 18% water content was used as a control.

The material was stored for 4 weeks. After this time, the grain was ground and pre-treated, as described in the Methods section.

The resulting material was used as a substrate in test fermentations in shake flask cultures using *S. cerevisiae *as the fermentation yeast. Samples were taken and the sugar and ethanol concentrations were monitored. Fermentations were run until a plateau concentration of ethanol had been reached, which usually occurred within 30 hours of the start of the fermentation. After 30 hours, the yields of gram ethanol per gram added wheat grains were determined using the maximum ethanol concentration determined for the according fermentation for the calculations. The ethanol yield from dry grains was 0.42 g ethanol/g grain (± 0.01 standard deviation (SD), *N *= 6). Unexpectedly, the yield from stored moist wheat grains was significantly enhanced (*P *< 0.001), to 0.48 g ethanol/g added grain in samples without biocontrol yeast (± 0.01 SD, *N *= 3), and 0.47 g ethanol/g added grain in samples with added biocontrol yeast (± 0.01 SD, *N *= 5). The difference between yields obtained from the inoculated and non-inoculated grain was not significant. These results imply that the biocontrol yeast does not negatively influence the ethanol yield, and that moist storage of the grain increases ethanol yield by more than 10%.

### Impact of the grain storage system on starch degradation

**Figure 1:**
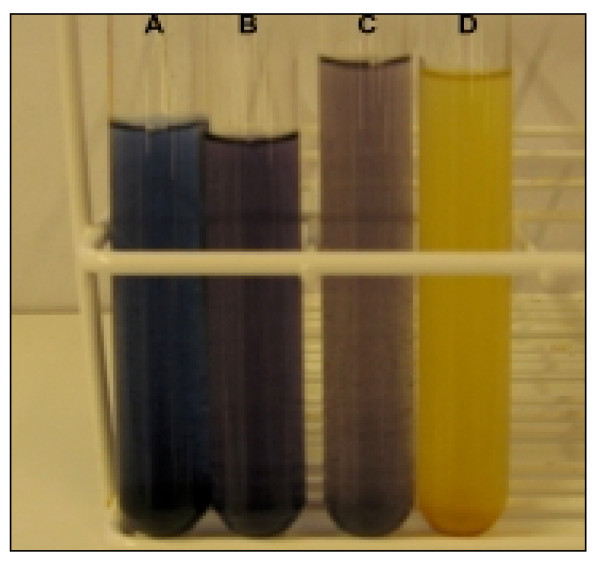
**Starch content screening with KI-I_2 _staining**. (a) Mash prepared from farm wheat grain before enzymatic pre-treatment. (b) Mash prepared from farm wheat grain after enzymatic pre-treatment with Stargen 001. (c) Mash prepared from stored moist wheat grain after enzymatic pre-treatment with Stargen 001. (d) Fermentation broth after fermentation.

To test starch degradation in the samples after the pre-treatment, mash samples were incubated with an I_2_-KI solution and the colour development was monitored. Mash samples obtained from dry grain were visibly darker than those from moist grain (Figure 1). This indicates a higher accessibility of the starch in moist grain to the enzymatic pre-treatment compared with the dry grain. Obviously a substantial amount of starch had not been degraded during the pre-treatments of the farm grain.

**Figure 2:**
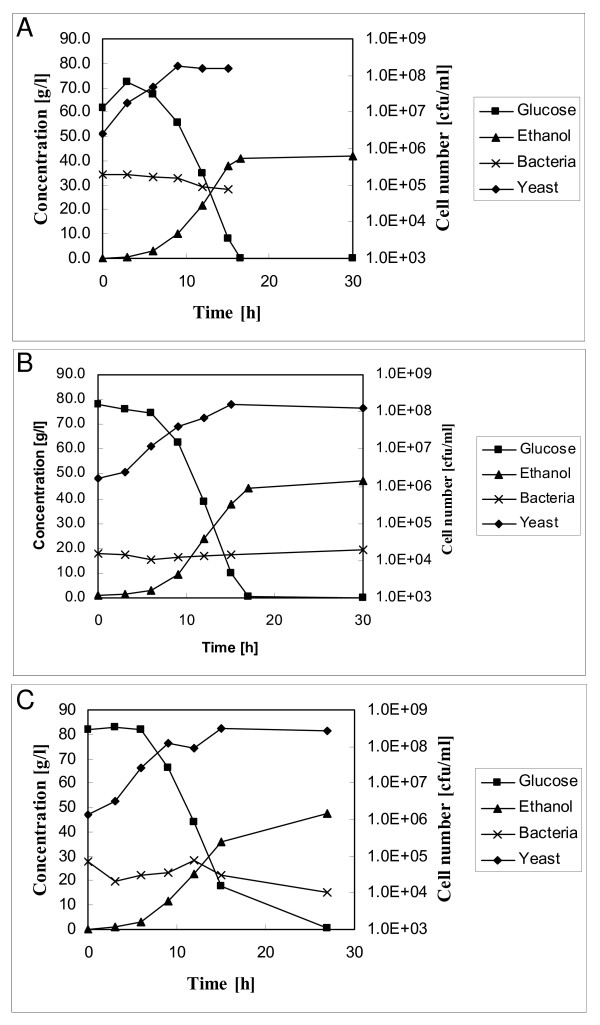
**Test fermentations of mashes prepared from differently stored grain**. Typical examples are shown. (a) Fermentation of mash obtained from stored farm grain. (b) Fermentation of mash obtained from stored moist grain in the presence of the biocontrol yeast, *P. anomala*.(c) Fermentation of mash obtained from stored moist grain without biocontrol yeast inoculation.

The more efficient starch degradation in the stored moist grain was also illustrated by the higher initial glucose concentrations in the test fermentation broth. The initial glucose concentrations in fermentations of stored moist grain, with or without biocontrol yeast, were around 80 g/L, compared with about 60 g/L in the fermentation medium obtained from farm grain (Figure 2). In the fermentations of the mash from farm grain, the sugar concentration increased during the first hours of cultivation, indicating a degradation of residual starch by glucoamylase, which was obviously still active in the fermentation. This increase in glucose concentration also indicated that there was starch left after the pre-treatment of stored dry grain. In fermentations of moist grains, with or without added biocontrol yeast, no increase or only a small initial increase in the glucose concentration was observed (Figure 2). The concentrations of free glucose dropped to 0 g/L between 12 and 15 hours of test fermentation in mashes from stored farm grain, and between 15 and 18 hours in those from stored moist grain. Some release of glucose from residual starch can be assumed even after the measured apparent glucose concentrations dropped to zero, as the plateaux in the ethanol concentration were reached somewhat later (Figure 2).

### Microbial populations in the test fermentations

Due to the high proportion of non-soluble particles in the substrate suspension, it was not possible to determine yeast biomass by gravimetric methods or optical density measurements. Therefore, yeast growth was monitored by plating dilutions of the fermentation broth on selective YPD medium. As shown in Figure 2, the number of yeast colony forming units (CFU) increased during fermentation, indicating that sugar consumption and ethanol production were connected to yeast growth. Cell numbers reached their maximum value some time before the plateau in the ethanol concentration was reached. In contrast to ethanol formation, no significant difference was found in the numbers of CFU in the different fermentations.

We also tested whether the use of biocontrol yeast had an impact on the composition of the yeast population in the test fermentations. DNA was isolated from 20 randomly selected colonies and a polymerase chain reaction (PCR)-fingerprint was generated and compared with the fingerprint of *P. anomala *J121 and that of the inoculated fermentation yeast (*S. cerevisiae*). The fingerprints of all tested isolates were identical to that of *S. cerevisiae *(results not shown). *P. anomala *was most probably already inactivated during the pre-treatment of the substrate. The substrate was not handled sterilely throughout the experiments and contaminating bacteria might thus influence the fermentations, so we also tested bacterial growth during the fermentation. Bacteria were present in the fermentation, but in low numbers compared with the yeast population (10^4^-10^5 ^bacterial CFU/ml of the fermentation liquid, that is, 10^5^-10^6^/g (dry weight) grain) (Figure 2). Moreover, their numbers did not increase during the fermentation, indicating that these bacteria were not actively growing. Probably the bacterial CFU were due to spores present on the grain and survived the pre-treatment. These results indicate that bacteria from the grain did not influence the ethanol production, independently of whether the grain was stored moist or dry.

### Influence of cellulases, hemicellulases and pectinases on ethanol yield

We also tested whether a treatment with a mixture of cellulases, hemicellulases and pectinases (CHP) could improve the ethanol yield. However, this treatment did not improve the ethanol yield, neither for dry nor moist stored grain. The high-performance liquid chromatography (HPLC) profiles of the fermentation broths from these treatments were almost identical to those obtained from material treated with Stargen 001 only. However, for the CHP-treated samples we found a peak in the HPLC chromatogram that did not disappear during the course of the fermentation. In contrast, in the samples without the additional enzyme treatment, a peak at the same retention time vanished towards the end of the fermentation. According to the standard curve, this peak represented maltose, which can be fermented to ethanol by several *S. cerevisiae *strains [13].

We further investigated the fermentation medium using a high-performance anion exchange chromatography coupled with pulsed amperometric detection (HPAE-PAD) analysis with a gradient method. This analysis showed that the peak was generated by both maltose and cellobiose. HPAE-PAD analysis showed that maltose and cellobiose were also present after fermentation in samples that were only treated with starch-degrading enzymes, however, the concentrations were below the lowest concentration of the standard (10 μM). In samples additionally treated with CHP, the amounts of maltose and cellobiose were higher compared with the fermentations of materials not treated with these enzymes. The concentrations of both disaccharides decreased during fermentation, indicating an assimilation of the maltose and/or an enzymatic degradation of both sugars during fermentation. Both GC200 and Multifect^® ^Pectinase FE have been shown to contain considerable β-glucosidase activity [14,15]. On the other hand, it has also been shown that an amendment of additional β-glucosidase to a GC200/Multifect^® ^Pectinase FE mixture can improve the degradation of cellulosic biomass [14]. However, in our experiments the maximum concentrations of cellobiose were below 0.3 g/L (results not shown), thus it does not seem to be relevant for increasing the ethanol yield from wheat grain. HPAE-PAD analysis also revealed the presence of small amounts of xylose, arabinose and galactose in the fermentation broths. Here concentrations were almost twice as high in CHP-treated samples compared with those treated only with Stargen 001. The amount of these sugars did not decrease during the fermentations in accordance with the stated inability of *S. cerevisiae *to ferment these sugars [13]. However, in all fermentations the concentrations of these sugars were always less than 1 g/L (results not shown), thus the potential impact of fermenting these sugars on the total ethanol yield from wheat grain seems to be insignificant.

## Discussion

In this study, we investigated whether airtight storage of moist wheat grain influences subsequent ethanol production from the material. Unexpectedly, the ethanol yields were more than 10% higher when using moist stored wheat instead of the dry material. The determined yield of about 0.47 g ethanol/g grain (dry weight) in our non-optimised control fermentations was higher than the 0.43 g usually obtained from dry grain by the Swedish ethanol industry . Thus, airtight storage of cereal grains, with considerably lower demand for process energy for storage of the material also substantially improved the ethanol fermentation. This effect was seen for stored moist material independently of whether a biocontrol yeast was added or not. Drying the grain apparently made the starch less accessible for the enzymes, as indicated by the higher starch content and the lower glucose concentrations detected after enzymatic pre-treatment. It has been demonstrated that the water content in starch has a strong influence on its network structure, with a high crystallisation and less susceptibility to enzyme degradation at low water content [16]. Airtight wet storage or ensilation has also been shown to improve the digestibility of cereal grain starch in animal feeding due to the activity of internal amylases and a more accessible structure of the starch granules [10,17]. It is also possible that phytic acid is degraded in moist grain due to endogenous and microbial activity [18]. Phytate can have a negative impact on starch degradation as it removes minerals from the mash, thus diminishing the activities of the starch-degrading enzymes [19]. Fermentation times and yeast growth were not substantially influenced by the storage method.

The microbial stability of the ethanol fermentation was not negatively affected by the presence of the biocontrol yeast in the grain storage. In all samples, the investigated yeast colonies were exclusively from the inoculated fermentation strain *S. cerevisiae *AEF1. The number of bacterial contaminants in the ethanol fermentation was low and there was no substantial difference between grains stored moist or dry, again indicating that airtight-stored grains can be used in ethanol production. No substantial yield improvement was obtained by the use of a mixture of CHP-enzymes. The fibre content of wheat is about 12% [20], which implies a certain potential for improving yield by the use of those enzymes. However, the fibre fraction seems to be resistant to enzymatic degradation and requires a thermochemical pre-treatment for its conversion into ethanol [14].

## Conclusion

Our results show that there is great potential for improving the efficiency of even firmly established processes like ethanol production from cereal grains, by using alternative methods for handling the material. Process energy consumes a major part of the energy gain when producing ethanol from grains [2], but it is obviously possible to decrease considerably the demand for process energy by an appropriate storage system and even to obtain an improved ethanol yield. Adding the biocontrol yeast *P. anomala *to the airtight system substantially improved the storage stability of the grain and did not impair ethanol production. These are important steps towards sustainable biofuel production.

## Methods

### Yeast strains and cultivation of micro-organisms

*Saccharomyces cerevisiae *AEF1, an isolate from a Swedish ethanol production plant was kindly provided by the Agroetanol company (Norrköping, Sweden). *Pichia anomala *J121 (CBS 100487) was used as the biopreservative yeast [4].

Yeasts were grown on YPD medium (20 g/L glucose, 20 g/L peptone and 10 g/L yeast extract, 16 g/L agar for solid medium) at 30°C. Selective YPD medium additionally contained 0.1 g/L chloramphenicol (Sigma-Aldrich Inc., St Louis, MO, USA) to suppress the growth of bacteria.

To study the presence of bacteria in the fermentations, a solid medium selective for bacteria was used, LB medium (10 g/L trypton, 5 g/L NaCl, 5 g/L yeast extract, 16 g/L agar and 0.1 g/L Delvocid [active compound natamycin, Gist-Brocades, Delft, The Netherlands]).

### Enzymes

For starch degradation, Stargen™ 001 (Danisco US Inc., Rochester, NY, USA), a mixture of alpha amylase and glucoamylase for industrial ethanol fermentations was used. The activity of the Stargen 001 enzyme mixture has been determined to be at least 456 granular starch hydrolysing units/g protein. GC 220 (Danisco US Inc., Rochester, NY, USA) was used as a cellulose and hemicellulose-degrading enzyme mixture, and Multifect^® ^Pectinase FE (Danisco US Inc., Rochester, NY, USA) was used for pectin degradation. The cellulase activity of GC 220 has been determined to be at least 6200 carboxymethylcellulose activity units (IU)/g protein. One IU unit is defined as the activity needed to liberate 1 μmol of reducing sugars in 1 minute. The pectinase activity of the Multifect^® ^Pectinase has been determined to be at least 145 IU pectinase/g protein. Enzyme activities were determined by the provider. All enzymes were a kind gift from Danisco US Inc., Genencor Division (Rochester, NY, USA).

### Wheat storage

The wheat grain was kindly provided by a local farmer (Anders Eriksson, Uppsala, Sweden). The farm grain (18% water content) was stored in a covered plastic barrel and used as a control. Moist wheat grain (30% water content) was obtained by re-moistening the dry grain and stored as 18-g portions in airtight reaction tubes with a simulated air leakage as previously described [4]. Biocontrol yeast was added to some of the tubes at a concentration of 10^5 ^cells/g wheat grain [21]. Moist grain was stored for 4 weeks before preparation of the fermentation mash. All storage occurred at room temperature.

### Preparation of a fermentation mash from wheat grains

Aliquots of 10 g of wheat grains (dry weight) were milled with a mixer (Braun kitchen machine) until the milled material could pass through a 1-mm screen. The resulting flour was mixed with water and the pH was adjusted to 5 with sulphuric acid. The final volume was 40 ml, which was poured into a 100-ml glass bottle. The suspension was gelatinised in a 100°C water bath for 25 minutes. Water was added to a volume of approximately 80 ml and the pH was readjusted to 5 if required. Subsequently, 25 μl of an amylase mixture (Stargen 001) was added and the bottles were incubated on a rotary shaker at 37°C and 100 rpm for 24 hours. A mixture of CHP-enzymes was added to the suspension of some of the samples before amylase incubation. This mixture contained GC 220 (3 mg protein/g grain dry weight) and Multifect^® ^Pectinase FE (0.1 mg protein/g grain dry weight). These amounts were calculated according to the supplier's recommendation to give a final enzyme concentration of 25 mg/g cellulose and 10 mg/g pectin, using the grain composition reported by Åman [20]. The samples with added CHP-enzymes were incubated at 37°C and 100 rpm for 72 hours; subsequently the samples were treated with amylases as described above.

After enzymatic pre-treatment, the bottles were filled to a volume of exactly 100 ml with water, the pH was adjusted to 5 and 1-ml sample was taken for sugar concentration determination.

### Analytical methods

#### Dry weight of wheat grains

The dry weight of wheat grain was determined by drying the grains in portions of 4 × 5 g wheat, in an oven at 80°C for 1 hour, followed by 15 hours at 105°C [22].

#### Starch analysis

Samples for starch analysis were taken from the fermentation mash. The samples were diluted 10 times. KI-I_2_-solution (0.1 sample volume; 20 g/L KI (Sigma-Aldrich, Steinheim, Germany) and 2 g/L I_2 _(Merck) was added to the samples [23], and the colour development was visually determined (an exact photometric quantification was not possible because the undissolved particles in the suspension disturbed the measurements). The colour change always occurred immediately after adding the KI-I_2_-solution.

#### Sugar and ethanol quantification

Glucose, maltose and ethanol were determined by HPLC as described by Fredlund *et al*. [24]. A mixture of glucose, maltose and ethanol at 1 g/L, 5 g/L, 10 g/L, 20 g/L and 50 g/L, respectively, was analysed to obtain calibration curves for these compounds. The glucose concentration was also determined by the enzymatic glucose oxidase-peroxidase (GOD-POD) method. Glucose oxidase was purchased from Sigma-Aldrich, horseradish peroxidase from Roche Diagnostics and ABTS (2,2'-azino-bis [3-ethylbenzthiazoline-6-sulphonic acid] di-ammonium salt) from Sigma-Aldrich. Standard curve and sample treatment were performed as described by Bergmeyer [25]. This method is specific for glucose and was used to validate the HPLC determinations. In HPLC chromatograms peaks can overlap and because of this give apparently higher concentrations of the analysed compounds. The glucose concentrations obtained with the GOD-POD method were indeed slightly smaller than those identified by HPLC. However, the differences were smaller than the standard deviations of both methods.

#### High-performance anion exchange coupled with pulsed amperometric detection

HPAE-PAD was used to measure mono- and disaccharides after enzymatic pre-treatment and after fermentation (Dionex Reference Library, 2006, ). The chromatography system used for mono and disaccharides was a Dionex (Sunnyvale, CA, USA) ICS-3000 system. The system consisted of a detector with a gold working electrode running in the integrated amperometry mode. The anion-exchange column used was a 2 × 250 mm analytical CarboPac PA1 column at 30°C. The waveform was carbohydrates standard quad potential (Dionex). All eluents were kept blanketed under helium pressure at all times to reduce carbonate build-up.

A gradient method was used for the determination of maltose and cellobiose where 100 mM NaOH without (eluent A) and with 200 mM sodium acetate (eluent B) were used as eluents, with a gradient from 0 to 85% B in 25 minutes at a flow rate of 0.25 ml/min. The standard curve for calibration was made with a mixture of maltose and cellobiose ranging from 10 to 100 μM in concentration.

Monosaccharides were separated with an isocratic method using 15 mM NaOH and a flow rate of 0.25 ml/min. Arabinose, galactose, glucose and xylose were identified in samples by comparison of retention times with standards of these sugars.

### Fermentation

*S. cerevisiae *was inoculated in a YPD medium and grown for 15 hours at 30°C and 150 rpm. Then 1 ml of this culture was added to 100 ml wheat grain mash in a 100-ml serum flask sealed by a rubber cork with a syringe needle through it and incubated at 30°C and 150 rpm. The test fermentations were run for 30 hours. Concentration of free glucose was directly tested during the fermentations using test stripes for glucose (Keto-Diabur-Test 5000, Roche, Mannheim, Germany) from 15 hours of cultivation until fermentation was finished. This was to make sure that no residual glucose was present when the fermentation was stopped.

### Microbial quantification

Samples from the test fermentations were diluted in steps of 10-fold dilutions and 10 μl of the dilutions were dropped on selective plates for yeast and bacteria, and incubated at room temperature for 24 hours. Colonies in the drops were counted and the numbers of yeast and bacteria per millilitre of fermentation broth was calculated.

### PCR-fingerprinting of yeast isolates

Yeast colonies from the fermentations were picked randomly from the quantification plates. Yeast DNA was isolated according to Liberal *et al*. [26] and PCR-fingerprints were generated as described earlier [12,27]. As controls, PCR-fingerprints were produced using genomic DNA of *S. cerevisiae *AEF1 and *P. anomala *J121 as templates.

### Statistics

To test if there were significant differences between the investigated storage methods, Student's t-test was performed with a significance level of 5% using Microsoft^® ^Excel 2000.

## Abbreviations

CFU: colony forming units; CHP: cellulase, hemicellulase and pectinase; GOD-POD: glucose oxidase-peroxidase; HPAE-PAD: high-performance anion exchange chromatography coupled with pulsed amperometric detection; HPLC: high-performance liquid chromatography; PCR: polymerase chain reaction; SD: standard deviation.

## Competing interests

The authors declare that they have no competing interests.

## Authors' contributions

VP performed the major part of the design of the project and experiments, the evaluation of results and writing the manuscript. AE was involved in the design of the study, performed most of the laboratory work and contributed to the evaluation of results and writing the manuscript. MS and JSt were involved in the design of the project, set up and partially performed the pre-treatment part and were involved in the evaluation of results and in writing the manuscript. KP designed the experiments for sugar analyses and contributed to the evaluation of results and writing the manuscript. JSc was involved in the design and coordination of the project, the evaluation of results and writing the manuscript. All authors read and approved the final manuscript.

## References

[B1] Pick E, Noren O, Nielsen V (1989). Energy Consumption and Input-output Relations of Field Operations.

[B2] Farrell AE, Plevin RJ, Turner BT, Jones AD, O'Hare M, Kammen DM (2006). Ethanol can contribute to energy and environmental goals. Science.

[B3] Passoth V, Fredlund E, Druvefors UÄ, Schnürer J (2006). Biotechnology, physiology and genetics of the yeast *Pichia anomala*. FEMS Yeast Res.

[B4] Petersson S, Schnürer J (1995). Biocontrol of mold growth in high-moisture wheat stored under airtight conditions by *Pichia anomala*, *Pichia guilliermondii*, and *Saccharomyces cerevisiae*. Appl Environ Microbiol.

[B5] Druvefors U, Jonsson N, Boysen ME, Schnürer J (2002). Efficacy of the biocontrol yeast *Pichia anomala *during long-term storage of moist feed grain under different oxygen and carbon dioxide regimens. FEMS Yeast Res.

[B6] Olstorpe M (2008). Feed grain improvement through biopreservation and bioprocessing. PhD thesis.

[B7] Kirk O, Borchert TV, Fuglsang CC (2002). Industrial enzyme applications. Curr Opin Biotechnol.

[B8] Nigam P, Singh D (1995). Enzyme and microbial systems involved in starch processing. Enzyme Microb Technol.

[B9] Maarel MJ van der, Veen B van der, Uitdehaag JC, Leemhuis H, Dijkhuizen L (2002). Properties and applications of starch-converting enzymes of the alpha-amylase family. J Biotechnol.

[B10] Rowe JB, Choct M, Pethick DW (1999). Processing cereal grains for animal feeding. Aust J Agric Res.

[B11] de Souza Liberal AT, Basilio AC, do Monte Resende A, Brasileiro BT, da Silva-Filho EA, de Morais JO, Simoes DA, de Morais MA (2007). Identification of *Dekkera bruxellensis *as a major contaminant yeast in continuous fuel ethanol fermentation. J Appl Microbiol.

[B12] Passoth V, Blomqvist J, Schnürer J (2007). *Dekkera bruxellensis *and *Lactobacillus vini *form a stable ethanol-producing consortium in a commercial alcohol production process. Appl Environ Microbiol.

[B13] Kurtzman CP, Fell JW (1998). The Yeasts, A Taxonomic Study.

[B14] Dien BS, Ximenes EA, O'Bryan PJ, Moniruzzaman M, Li XL, Balan V, Dale B, Cotta MA (2008). Enzyme characterization for hydrolysis of AFEX and liquid hot-water pretreated distillers' grains and their conversion to ethanol. Bioresourc Technol.

[B15] Kabel MA, Maarel MJEC van der, Klip G, Voragen AGJ, Schols HA (2006). Standard assays do not predict the efficiency of commercial cellulase preparations towards plant materials. Biotechnol Bioeng.

[B16] Bayer RK, Cagiao ME, Calleja FJB (2006). Structure development in amorphous starch as revealed by X-ray scattering: Influence of the network structure and water content. J Appl Polym Sci.

[B17] Perttilä S, Valaja J, Partanen K, Jalava T, Kiiskinen T, Palander S (2001). Effects of preservation method and beta-glucanase supplementation on ileal amino acid digestibility and feeding value of barley for poultry. Br Poult Sci.

[B18] Lyberg K, Olstorpe M, Passoth V, Schnürer J, Lindberg JE (2008). Biochemical and microbial properties of a cereal mix fermented with whey, wet wheat distiller's grain or water at different temperatures. Anim Feed Sci Technol.

[B19] Shetty JK, Paulson B, Pepsin M, Chotani G, Dean B, Hruby M (2008). Phytase in fuel ethanol production offers economical and environmental benefits. Int Sugar J.

[B20] Åman P (1987). Analys och Kemisk Sammansättning av Svensk Spannmål. Fakta Husdjur 3.

[B21] Druvefors UÄ, Passoth V, Schnürer J (2005). Nutrient effects on biocontrol of *Penicillium roqueforti *by *Pichia anomala *J121 during airtight storage of wheat. Appl Environ Microbiol.

[B22] Eaton AD, Clesceri LS, Greenberg AE (1995). Standard Methods, for the Examination of Water and Wastewater.

[B23] Schmieder RL, Keeney PG (1980). Characterization and quantification of starch in cocoa beans and chocolate products. J Food Sci.

[B24] Fredlund E, Blank LM, Schnürer J, Sauer U, Passoth V (2004). Oxygen- and glucose-dependent regulation of central carbon metabolism in *Pichia anomala*. Appl Environ Microbiol.

[B25] Bergmeyer HU (1974). Methods of Enzymatic Analysis.

[B26] Liberal ATD, da Silva EA, de Morais JOF, Simoes DA, de Morais MA (2005). Contaminant yeast detection in industrial ethanol fermentation must by rDNA-PCR. Lett Appl Microbiol.

[B27] Olstorpe M, Lyberg K, Lindberg JE, Schnürer J, Passoth V (2008). Population diversity of yeasts and lactic acid bacteria in pig feed fermented with whey, wet wheat distillers' grains, or water at different temperatures. Appl Environ Microbiol.

